# Waterborne Lead Exposure Induces Hepatic Oxidative Stress and Transcriptomic Responses in Pufferfish (*Takifugu obscurus*)

**DOI:** 10.3390/antiox15070827

**Published:** 2026-06-30

**Authors:** Shengli Fu, Kun Qian, Tuo Yao, Jie Lu, Lingtong Ye, Jianmin Ye

**Affiliations:** 1 Key Laboratory of Aquatic Product Processing, Key Laboratory of South China Sea Fishery Resources Exploitation & Utilization, Ministry of Agriculture and Rural Affairs, South China Sea Fisheries Research Institute, Chinese Academy of Fishery Sciences, Guangzhou 510300, China; fushengli@scsfri.ac.cn (S.F.);; 2 Sanya Tropical Fisheries Research Institute, Sanya 572426, China; 3 Guangzhou Key Laboratory of Subtropical Biodiversity and Biomonitoring, Guangdong Provincial Engineering Technology Research Center for Environmentally-Friendly Aquaculture, School of Life Sciences, South China Normal University, Guangzhou 510631, China

**Keywords:** antioxidant enzymes, hepatotoxicity, lead exposure, RNA-seq, *Takifugu obscurus*

## Abstract

Lead (Pb) is a persistent aquatic pollutant that disrupts redox homeostasis in fish. This study investigated hepatic Pb accumulation, reactive oxygen species (ROS) production, antioxidant responses, lipid peroxidation, and transcriptomic alterations in juvenile pufferfish (*Takifugu obscurus*) exposed to waterborne Pb. Juvenile pufferfish were exposed to 5.98 mg/L waterborne Pb, corresponding to 10% of the 96 h LC_50_, for 96 h. Liver, blood, and hepatocyte samples were collected at 0, 12, 24, 48, and 96 h, with four biological replicates at each sampling time point. Hepatic Pb accumulation increased over time and reached the highest level at 96 h. ROS levels in blood cells and hepatocytes increased rapidly and peaked at 12 h. Superoxide dismutase (SOD) and catalase (CAT) activities showed early activation followed by late suppression, whereas glutathione peroxidase (GSH-Px) displayed partial adaptive recovery. Malondialdehyde (MDA) content increased progressively and reached approximately 2.8-fold of the control level at 96 h, indicating persistent lipid peroxidation. RNA-seq analysis identified 167, 460, 1398, and 2580 differentially expressed genes at 12, 24, 48, and 96 h, respectively. Enrichment, temporal trend, and weighted gene co-expression analyses indicated that Pb exposure shifted hepatic responses from early redox regulation to later metabolic adaptation, protein processing in the endoplasmic reticulum, proteasome function, and oxidative phosphorylation. qRT-PCR validation of 12 hub genes supported the RNA-seq results. These findings provide integrated biochemical and transcriptomic evidence for oxidative-stress-mediated hepatic toxicity in pufferfish exposed to waterborne Pb.

## 1. Introduction

Heavy metals are persistent environmental pollutants that threaten aquatic ecosystems, fish health, and seafood safety worldwide [1,2,3]. Lead (Pb) is a ubiquitous and highly toxic contaminant released into aquatic environments through industrial discharge, agricultural runoff, mining activities, battery production, and urban wastewater [1,2,4]. Because Pb is not biodegradable, it can persist in water and sediment and can be absorbed by fish through the gills, digestive tract, and body surface. Accumulated Pb can then interfere with physiological homeostasis, immune function, tissue integrity, and growth performance, making fish sensitive sentinel organisms for assessing aquatic heavy metal pollution [4,5,6,7,8].

A central mechanism of Pb toxicity is the disruption of redox homeostasis. Pb exposure can promote excessive reactive oxygen species (ROS) generation, inhibit sulfhydryl-containing antioxidant molecules, interfere with antioxidant enzyme systems, and induce oxidative damage to lipids, proteins, and nucleic acids [4,9,10,11,12,13]. Recent reviews further emphasize that oxidative stress biomarkers, including ROS, superoxide dismutase (SOD), catalase (CAT), glutathione peroxidase (GSH-Px), and malondialdehyde (MDA), are widely used to evaluate heavy metal toxicity in fish [9,10,11]. When ROS production exceeds antioxidant capacity, lipid peroxidation products such as MDA accumulate, reflecting membrane damage and progressive oxidative injury [11,12,13].

The liver is a major organ responsible for metabolism, detoxification, antioxidant defense, and xenobiotic biotransformation in fish and is therefore particularly susceptible to heavy metal-induced injury [14,15,16,17]. In addition to classical biochemical indicators, transcriptomic analysis provides an effective approach for identifying molecular responses underlying metal-induced hepatotoxicity, including antioxidant defense, mitochondrial function, endoplasmic reticulum stress, proteasomal degradation, apoptosis, lipid remodeling, and energy metabolism [18,19,20,21]. Integrating biochemical markers with RNA-seq data can therefore provide a more comprehensive understanding of the temporal regulatory mechanisms by which fish respond to Pb-induced oxidative stress.

Pufferfish (*Takifugu obscurus*) is an economically important freshwater and brackish-water species cultured in East Asia [22,23]. However, information on hepatic antioxidant responses and transcriptomic regulation in pufferfish under Pb exposure remains limited. In particular, few studies have combined Pb bioaccumulation, cellular ROS detection, antioxidant enzyme assays, lipid peroxidation, time-series RNA-seq, temporal expression trend analysis, and co-expression network analysis to characterize the acute hepatic response of this species to waterborne Pb. To address this knowledge gap, juvenile pufferfish were exposed to a sublethal concentration of waterborne Pb for 96 h. Hepatic Pb accumulation, ROS production in blood cells and hepatocytes, antioxidant enzyme activities, MDA content, transcriptomic profiles, temporal expression trends, weighted gene co-expression networks, and qRT-PCR validation of hub genes were analyzed. To our knowledge, this is the first study to integrate these biochemical and transcriptomic approaches to clarify Pb-induced hepatic oxidative stress and transcriptomic remodeling in *T. obscurus*, providing evidence relevant to environmental toxicology and antioxidant biology.

## 2. Materials and Methods

### 2.1. Materials

Healthy juvenile male pufferfish with an average body weight of 20.3 ± 2.4 g and body length of 7.4 ± 1.1 cm were purchased from Jingyan Aquaculture Co., Ltd. (Guangzhou, China). Before the experiment, fish were acclimated for two weeks in 100 L recirculating plastic tanks (10 fish per tank) and fed twice daily with a commercial diet (42.0% protein, 3.0% fat, 6.0% fiber, and 16.0% ash; Dabeinong Group, Beijing, China). Only healthy fish showing normal swimming and feeding behavior and no visible body surface injury were used. Water quality was maintained at 24–26 °C, dissolved oxygen ≥ 7.0 mg/L, and pH 7.5–7.7 during acclimation. Fish were fasted for 24 h before sampling.

### 2.2. Exposure Treatment and Sample Collection

A 96 h acute toxicity test was conducted to determine the median lethal concentration (LC_50_) of waterborne Pb using Pb(NO_3_)_2_ (Tianjin Damao Chemical Reagent Co., Ltd., Tianjin, China) as the Pb source, according to previously described procedures [24]. Based on preliminary experiments, the 96 h LC_50_ of waterborne Pb for pufferfish was determined to be 59.8 mg/L as elemental Pb. The sublethal exposure concentration was calculated as 10% of the 96 h LC_50_ and was therefore set at 5.98 mg/L as elemental Pb.

Before Pb exposure, four fish were randomly collected from four tanks, with one fish sampled from each tank, as the control group (Control, *n* = 4). The remaining fish were exposed to 5.98 mg/L waterborne Pb for 96 h, and four fish were sampled at 12, 24, 48, and 96 h, respectively (Pb_12 h, Pb_24 h, Pb_48 h, and Pb_96 h sampling time points; *n* = 4 per time point). At each sampling time point, fish were deeply anesthetized with 0.05% MS-222 (Aladdin, Shanghai, China) until loss of reflex response and then euthanized for blood and liver collection. Blood and liver samples were collected immediately for ROS measurement, Pb accumulation analysis, antioxidant enzyme assays, MDA determination, RNA sequencing, and qRT-PCR validation. No sampled fish were returned to the tanks, and all fish included in the exposure experiment were euthanized at their assigned sampling time points.

### 2.3. Pb Accumulation

Liver samples from each group were analyzed for Pb concentration. Samples were homogenized and digested with 4 mL HNO_3_ for 1 h and then processed in a microwave digestion apparatus (Shanghai QiYao Co., Ltd., Shanghai, China) using the following program: heating to 120 °C over 5 min (held for 5 min), 150 °C over 5 min (held for 10 min), and 190 °C over 5 min (held for 20 min). After digestion, samples were degassed for 2 h and diluted to 50 mL with ultrapure water. Pb concentrations were measured using inductively coupled plasma mass spectrometry (ICP-MS) after calibration with a standard curve, following the method described by Habte et al. [25]. The accumulation rate was calculated as the increase in Pb concentration per unit time at each sampling point.

### 2.4. ROS Measurements

ROS levels were monitored using the cell-permeant probe 2′,7′-dichlorofluorescein diacetate (DCFH-DA; Beyotime, Shanghai, China), which is deacetylated intracellularly and oxidized by ROS to form fluorescent DCF, following a previously described fluorescence-based method [26]. Blood cell or hepatocyte suspensions (200 μL) were diluted with anticoagulant solution to a final concentration of 1 × 10^6^ cells/mL. DCFH-DA was added to a final concentration of 10 μM, and samples were incubated in the dark at room temperature for 30 min. After washing with phosphate-buffered saline to remove excess probe, fluorescence was analyzed using a Becton–Dickinson FACS Calibur flow cytometer (Becton, Dickinson and Company, Franklin Lakes, NJ, USA). Forward and side scatter parameters were used to exclude debris and aggregates, and 10,000 cells were analyzed per sample. ROS production was expressed as the mean fluorescence intensity of DCF.

### 2.5. Enzyme Activities Analysis

Liver homogenates were prepared by mixing liver tissue with physiological saline at a ratio of 1:9 (*w*/*v*), followed by mechanical homogenization on ice for 3–5 min. Homogenates were centrifuged at 4000× *g* for 10 min at 4 °C, and the supernatants were collected. The activities of SOD, CAT, and GSH-Px, as well as MDA content, were determined using commercial kits from Nanjing Jiancheng Bioengineering Institute (Nanjing, China), including SOD assay kit (Cat. No. A001-3-2), CAT assay kit (Cat. No. A007-1-1), GSH-Px assay kit (Cat. No. A005-1-2), and MDA assay kit (Cat. No. A003-1-2), according to the manufacturer’s instructions.

SOD activity was assessed using the WST-1 (water-soluble tetrazolium-1) method at 450 nm [27]. One unit of SOD activity was defined as the amount of enzyme required to inhibit the reduction in WST-1 by superoxide anion by 50%. CAT activity was measured by monitoring H_2_O_2_ degradation at 405 nm [28]. One unit of CAT activity was defined as the amount of enzyme required to decompose 1 μmol/L H_2_O_2_. GSH-Px activity was determined by monitoring reduced glutathione consumption according to the manufacturer’s protocol [29]. MDA content was determined using the thiobarbituric acid reaction at 532 nm [30]. Briefly, MDA in the liver homogenate reacted with thiobarbituric acid under acidic and high-temperature conditions to form a colored MDA-TBA adduct. Absorbance was measured at 532 nm, and MDA content was calculated according to the standard curve and normalized to protein concentration.

### 2.6. RNA Isolation, cDNA Library Preparation, and Sequencing

Total RNA was isolated from liver samples using TRIzol reagent (Invitrogen, Waltham, MA, USA). RNA integrity was assessed by 1% agarose gel electrophoresis, and RNA purity was evaluated using a NanoPhotometer^®^ spectrophotometer (IMPLEN GmbH, Munich, Germany). RNA quality and quantity were further confirmed using a NanoDrop 2000 spectrophotometer (Thermo Fisher Scientific, Waltham, MA, USA), and samples with RNA integrity number ≥ 7.0 and A_260_/A_280_ = 1.8–2.1 were used for library construction.

Sequencing libraries were constructed using the NEBNext^®^ Ultra™ RNA Library Prep Kit for Illumina^®^ (New England Biolabs, Ipswich, MA, USA) following the manufacturer’s protocol. mRNA was purified using poly-T oligo-attached magnetic beads, fragmented using divalent cations in NEBNext First Strand Synthesis Reaction Buffer (5×), and reverse transcribed into first-strand cDNA using random hexamer primers and M-MuLV Reverse Transcriptase (RNase H^−^). Second-strand cDNA was synthesized using DNA polymerase I and RNase H. After end repair, adaptor ligation, size selection, and PCR amplification, library quality was assessed using an Agilent Bioanalyzer 2100 system. Libraries were sequenced on an Illumina HiSeq platform to generate paired-end reads, and transcript abundance was quantified as FPKM using RSEM (version 1.2.15) [31].

### 2.7. Differential Gene Expression and Enrichment Analyses

Differential expression analysis between sampling time points was performed using the DESeq R package (version 1.10.1), which applies a negative binomial model to RNA-seq count data [32]. *p*-values were adjusted using the Benjamini–Hochberg false-discovery-rate method [33]. Genes with adjusted *p* < 0.05 and an absolute fold change ≥ 2 were considered differentially expressed genes (DEGs).

DEGs were annotated using the Gene Ontology (GO; http://geneontology.org, accessed on 29 March 2026) and Kyoto Encyclopedia of Genes and Genomes (KEGG; http://www.genome.jp/kegg, accessed on 29 March 2026) databases [34,35]. GO and KEGG enrichment analyses were performed using a hypergeometric test, with adjusted *p* < 0.05 and rich factor > 1.5 as thresholds. The top 20 significantly enriched terms or pathways were selected for visualization.

### 2.8. Temporal Expression Trend Analysis

Temporal expression trends of DEGs were analyzed using Short Time-series Expression Miner (STEM v1.3.12) with default parameters (maximum unit change in model profiles = 1 and minimum correlation coefficient = 0.7) [36]. The statistical significance of DEG enrichment in each profile relative to the expected gene number was calculated using Fisher’s exact test, and profiles with *p* < 0.05 were considered significant. For each significant profile, the gene expression trend was summarized, and KEGG pathway enrichment analysis was performed (adjusted *p* < 0.05) to identify core functional pathways. Core DEGs in each profile were screened based on FPKM > 10 and fold change > 4.

### 2.9. Weighted Gene Co-Expression Network Construction

Weighted gene co-expression network analysis (WGCNA) was performed using the WGCNA package (v1.47) in R [37]. Genes with FPKM < 0.3 in ≥50% of samples were excluded before network construction. Co-expression modules were constructed using the block-wise Modules function with the following parameters: power = 10, TOM Type = unsigned, merge Cut Height = 0.25, and min Module Size = 50.

### 2.10. qRT-PCR Verification

Twelve DEGs (*psmd1*, *psmd7*, *psmc4*, *psma5*, *atp6v0b*, *tcirg1b*, *sec31a*, *sar1b*, *uggt2*, *atp6v1e1b*, *atp6v1g1*, *atp6v1c1*) were selected for qRT-PCR validation. These target genes were selected from the transcriptome dataset generated in the present study. RNA samples were the same as those used for library construction. Primers (Table 1) were designed using Primer 5.0 (Premier Biosoft International, Palo Alto, CA, USA) and synthesized by Tsingke Biological Technology Co., Ltd. (Beijing, China) with *β-actin* (GenBank Accession No. EU871643.1) and *gapdh* (GenBank Accession No. AB704200.1) as dual reference genes. qRT-PCR was performed on an ABI 7500 Real-Time PCR system (Applied Biosystems, Fosrer City, CA, USA) using SYBR Green dye (TaKaRa Bio Inc., Kusatsu, Japan). The 20 μL reaction mixture contained 3 μL of 1:10 diluted cDNA, 10 μL of 2× TaKaRa Ex Taq™ SYBR premix, 2 μL of each primer (10 μM), 0.4 μL of ROX Reference Dye II (Takara, Japan), and 2.6 μL of ddH_2_O. The PCR program was: 94 °C for 30 s, followed by 40 cycles of 95 °C for 5 s and 60 °C for 30 s, with a final melting curve analysis. Each sample was analyzed in triplicate. The relative expression levels of the target genes were calculated as fold change by normalization to *β-actin*. The results were analyzed using the 2^−ΔΔCt^ method after normalization to the geometric mean of *β-actin* and *gapdh* expression [38].

### 2.11. Statistical Analysis

Data are presented as mean ± standard deviation (SD). For biochemical and physiological measurements, each sampling time point included four biological replicates (*n* = 4), with each replicate corresponding to one individual fish sampled from a different tank. Normality was assessed using the Shapiro–Wilk test, and homogeneity of variance was evaluated using Levene’s test. For datasets satisfying the assumptions of normality and homogeneity of variance, differences among sampling time points were analyzed using one-way ANOVA followed by Duncan’s multiple range test. When the assumption of homogeneity of variance was not met, Welch’s one-way ANOVA followed by the Games–Howell post hoc test was applied. Because the hepatic Pb concentration data did not satisfy the assumption of homogeneity of variance, these data were analyzed using Welch’s one-way ANOVA followed by the Games–Howell test. Statistical significance was set at *p* < 0.05. Graphs were generated using GraphPad Prism 9, and statistical analyses were performed using SPSS 26.0.

## 3. Results

### 3.1. Hepatic Pb Accumulation

**Figure 1:**
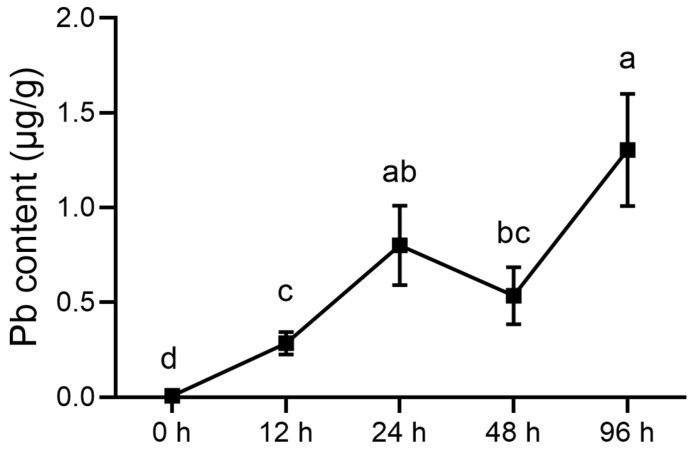
Pb accumulation in the liver of pufferfish during 96 h of waterborne Pb exposure. Data are presented as mean ± SD (*n* = 4). Different letters indicate significant differences among sampling time points (*p* < 0.05).

Hepatic Pb concentration differed significantly among sampling time points [Welch’s ANOVA: F(4, 8.06) = 66.19, *p* < 0.001]. Compared with the 0 h sampling time point, hepatic Pb concentration increased significantly at 12, 24, 48, and 96 h. It increased from 12 to 24 h, showed a transient decrease at 48 h, and reached the highest mean level at 96 h. Games–Howell multiple comparisons showed that the 12 h value was significantly lower than those at 24 and 96 h but did not differ significantly from that at 48 h. The 24 h value did not differ significantly from those at 48 or 96 h, whereas the 48 h value was significantly lower than that at 96 h. These findings indicate an overall increase in hepatic Pb accumulation during waterborne Pb exposure, accompanied by a transient fluctuation at 48 h (Figure 1).

### 3.2. ROS Production

**Figure 2:**
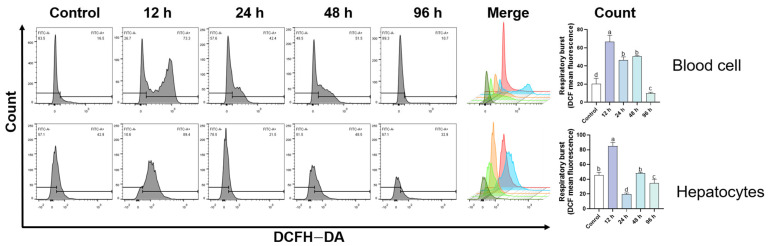
Reactive oxygen species (ROS) production in blood cells and hepatocytes of pufferfish during 96 h of Pb exposure. The gray histograms show DCF fluorescence distributions at each sampling time point, and the overlaid colored curves in the Merge panels correspond to Control, 12 h, 24 h, 48 h, and 96 h. Bar graphs show the mean DCF fluorescence intensity. Data are presented as mean ± SD (*n* = 4). Different letters indicate significant differences among sampling time points (*p* < 0.05).

ROS production in both blood cells and hepatocytes increased rapidly after Pb exposure and peaked at 12 h, reaching approximately 1.8-fold and 2.1-fold of the control levels, respectively (*p* < 0.05; Figure 2). Thereafter, ROS levels declined gradually. Blood-cell ROS remained significantly higher than the control level during 24–48 h and returned close to the control level at 96 h. Hepatocyte ROS also decreased after the 12 h peak and approached the control level during the later exposure period.

### 3.3. Antioxidant Enzyme Activities and MDA Content

**Figure 3:**
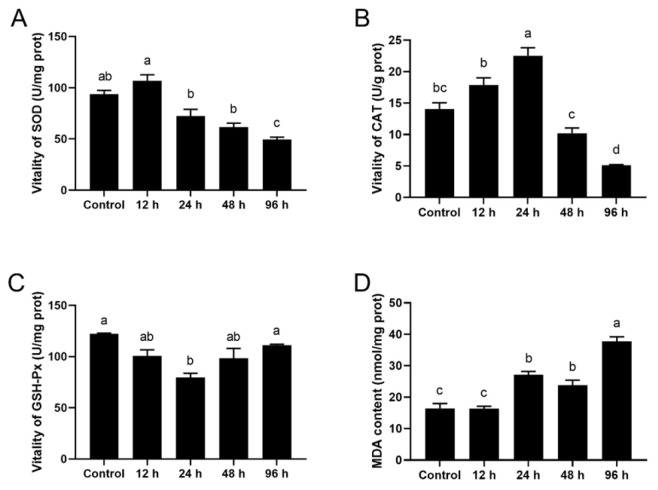
Effects of Pb exposure on antioxidant enzyme activities and malondialdehyde (MDA) content in the liver of Pufferfish. (**A**) Superoxide dismutase (SOD); (**B**) catalase (CAT); (**C**) glutathione peroxidase (GSH-Px); and (**D**) MDA content. Data are presented as mean ± SD (*n* = 4). Different letters indicate significant differences among sampling time points (*p* < 0.05).

Antioxidant enzyme activities and MDA content in the liver of pufferfish after Pb exposure are shown in Figure 3. Compared with the control group, SOD activity increased significantly at 12 h and then gradually declined (1.4-fold of the control *p* < 0.05), reaching a significantly lower level at 96 h (0.6-fold, *p* < 0.05) (Figure 3A). CAT activities increased significantly at both 12 and 24 h (1.3-fold and 1.2-fold of the control, respectively, *p* < 0.05), followed by a gradual decrease, and was significantly lower than the control level at 96 h (0.5-fold, *p* < 0.05) (Figure 3B). GSH-Px activity decreased at 24 h (0.7-fold of the control, *p* < 0.05), gradually recovered thereafter, and returned to the control level at 96 h (*p* > 0.05) (Figure 3C). MDA content increased progressively with exposure time and was approximately 2.8-fold higher than the control level at 96 h (*p* < 0.05). These results indicate an early antioxidant response followed by late oxidative damage and persistent lipid peroxidation (Figure 3D).

### 3.4. Transcriptomic Analysis

#### 3.4.1. Overview of RNA Transcriptome Profile

Twenty libraries were constructed using liver samples from four biological replicates at each sampling time point (Control, Pb_12 h, Pb_24 h, Pb_48 h, and Pb_96 h). After filtering low-quality reads (Q < 20), a total of 140.73 Gb of clean data were obtained, with Q20 ≥ 97.31% and Q30 ≥ 95.21% for all samples (Table S1), indicating high sequencing quality. Functional annotation of transcripts was performed using the NR, EggNOG, Swiss-Prot, Pfam, GO, and KEGG databases (Table S2, Figure S1).

#### 3.4.2. Identification of DEGs

**Figure 4:**
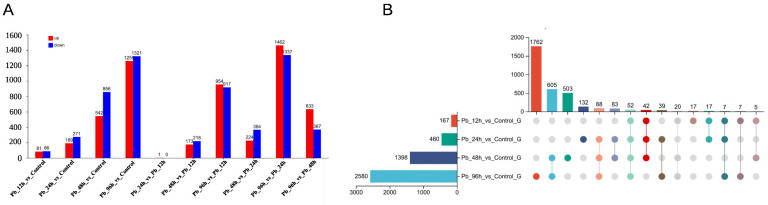
Differentially expressed genes (DEGs) in the liver of pufferfish under Pb exposure. (**A**) Numbers of upregulated and downregulated DEGs in each comparison. Red indicates upregulation and blue indicates downregulation. (**B**) Upset plot showing shared and unique DEGs among the four comparisons.

The number of DEGs increased significantly with exposure time: 167 DEGs (81 upregulated, 86 downregulated) in Pb_12 h vs. Control; 460 DEGs (189 upregulated, 271 downregulated) in Pb_24 h vs. Control; 1398 DEGs (542 upregulated, 856 downregulated) in Pb_48 h vs. Control; and 2580 DEGs (1259 upregulated, 1321 downregulated) in Pb_96 h vs. Control (Figure 4A). A total of 52 common DEGs were shared across all four comparison groups (Figure 4B), suggesting a core hepatic transcriptional response to Pb exposure.

**Figure 5:**
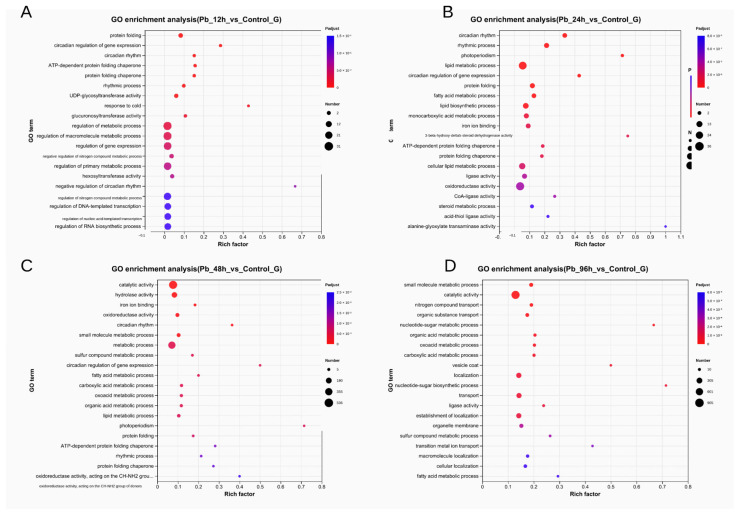
Gene Ontology (GO) enrichment analysis of DEGs in Pb_12 h vs. Control (**A**), Pb_24 h vs. Control (**B**), Pb_48 h vs. Control (**C**), and Pb_96 h vs. Control (**D**). The y-axis indicates GO terms, and the x-axis indicates the rich factor, defined as the ratio of DEGs enriched in a given GO term to all genes annotated to that term. Dot size represents the number of DEGs, and color indicates the adjusted *p*-value.

GO enrichment analysis revealed dynamic functional shifts in DEGs over time. In Pb_12 h vs. Control, DEGs were mainly enriched in protein binding, regulation of metabolic process, and regulation of gene expression (Figure 5A). In Pb_24 h vs. Control, enriched terms included lipid metabolic process, oxidoreductase activity, and fatty acid metabolic process (Figure 5B). In Pb_48 h vs. Control, DEGs were enriched in catalytic activity, small molecule metabolic process, and oxidoreductase activity (Figure 5C). In Pb_96 h vs. Control, dominant enriched terms included small molecule metabolic process, catalytic activity, and organic acid metabolic process (Figure 5D). These results indicate that Pb exposure induced a transition from early redox-related responses to later metabolic remodeling in the liver.

#### 3.4.3. Temporal Expression Trends During Pb Exposure

**Figure 6:**
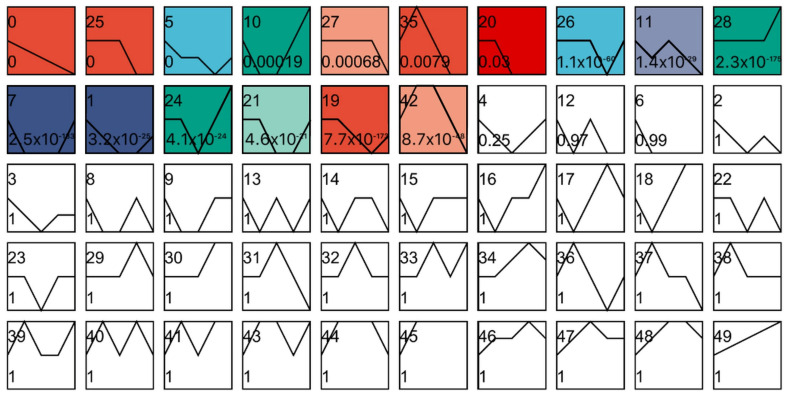
Temporal expression trends during Pb stress revealed by Short Time-series Expression Miner (STEM) analysis. Each box indicates a model profile, and the colored profiles shown are significant. The number in each box indicates the profile order, and the *p*-value indicates the significance of DEG enrichment in that profile.

**Figure 7:**
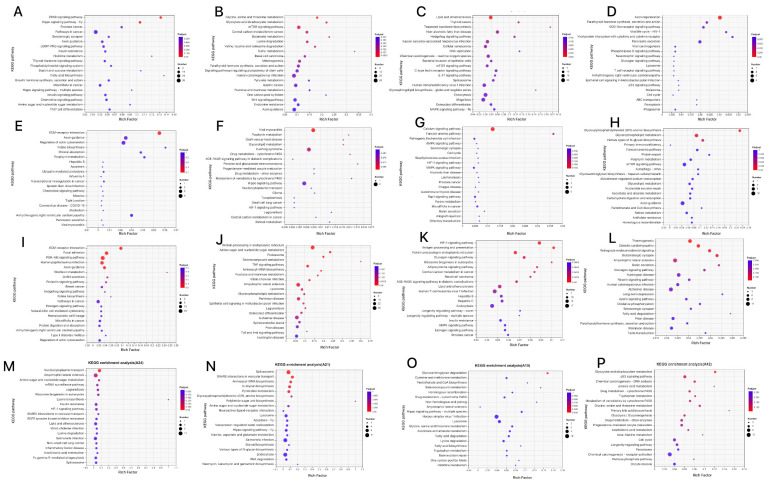
Top 20 Kyoto Encyclopedia of Genes and Genomes (KEGG) pathways enriched in genes assigned to STEM profiles 0 (**A**), 25 (**B**), 5 (**C**), 10 (**D**), 27 (**E**), 35 (**F**), 20 (**G**), 26 (**H**), 11 (**I**), 28 (**J**), 7 (**K**), 1 (**L**), 24 (**M**), 21 (**N**), 19 (**O**), and 42 (**P**).

STEM analysis identified 16 statistically significant temporal expression profiles of DEGs (Figure 6). These profiles reflected distinct gene expression trajectories across the Pb exposure period. KEGG enrichment of genes assigned to significant profiles further revealed pathway-specific temporal responses (Figure 7). The profile 0 genes were involved in PPAR and Hippo signaling pathways (Figure 7A). The profile of 25 genes were involved in mTPR signaling pathway, Glycin, serine and threonine metabolism and Glyoxylate and dicarboxylate metabolism (Figure 7B). The profile 5 genes were involved in Hedgehog, mTOR, C-type lectin receptor, IL-17, and MAPK signaling pathways (Figure 7C). The profile 10 genes were involved in Axon regeneration, lysosome, and Phagosoma (Figure 7D). The profile 27 genes were involved in ECM-receptor interaction, Apoptosis, and Chemokine signaling pathway (Figure 7E). The profile 35 genes were involved in Glycerolipid metabolism, Drug metabolism, and Hippo signaling pathways (Figure 7F). The profile 20 genes were involved in Calcium, AMPK and MAPK signaling pathway (Figure 7G). The profile 26 genes were involved in Glycerophospholipid metabolism and the mTOR signaling pathway (Figure 7H). The profile 11 genes were involved in ECM-receptor interaction, PI3K-Akt signaling pathway, and Focal adhesion (Figure 7I). The profile 28 genes were involved in protein processing in the endoplasmic reticulum, proteasome, and TNF signaling pathway (Figure 7J). The profile 7 genes were involved in the HIF-1 signaling pathway, Antigen processing and presentation (Figure 7K). The profile 1 genes were involved in Thermogenesis (Figure 7L). The profile 24 genes were involved in Nucleocytoplasmic transport, Lysine biosynthesis and degradation (Figure 7M). The profile 21 genes were involved in the Spliceosome, lysosome, and apoptosis (Figure 7N). The profile 19 genes were involved in Glycosaminoglycan degradation, Selenocompound and Trytophan metabolism (Figure 7O). The profile 42 genes were involved in P53 signaling pathway, cell cycle, and peroxisome (Figure 7P).

#### 3.4.4. Weighted Gene Co-Expression Network Analysis

**Figure 8:**
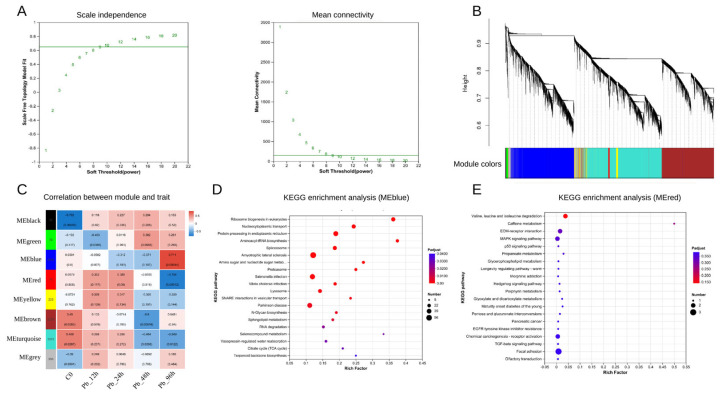
Identification of hub modules associated with Pb exposure in pufferfish by WGCNA. (**A**) Scale-free topology analysis is used to determine the soft-thresholding power. (**B**) Hierarchical clustering dendrogram of co-expressed genes. (**C**) Module-trait correlation heatmap. (**D**) Top 20 KEGG pathways enriched in genes from the MEblue module. (**E**) Top 20 KEGG pathways enriched in genes from the MEred module.

WGCNA identified co-expression modules associated with Pb exposure (Figure 8). A scale-free topological network was constructed with a soft-thresholding power of 10 (Figure 8A). Hierarchical clustering grouped highly co-expressed genes into distinct color-coded modules (Figure 8B). Module-trait correlation analysis revealed that the MEred module showed a significant positive correlation with Pb exposure, while the MEblue module exhibited a notable negative correlation (Figure 8C).

KEGG enrichment analysis was performed for hub modules to clarify their functions. Genes in the MEblue module were mainly enriched in protein processing in the endoplasmic reticulum, proteasome, and oxidative phosphorylation pathways (Figure 8D). Genes in the MEred module were enriched in pathways such as valine, leucine, and isoleucine degradation, ECM—receptor interaction, and MAPK signaling (Figure 8E). These module-level results suggest that Pb exposure coordinately affected protein homeostasis, energy metabolism, extracellular matrix signaling, and stress-related pathways.

#### 3.4.5. Hub Gene Network and qRT-PCR Validation

**Figure 9:**
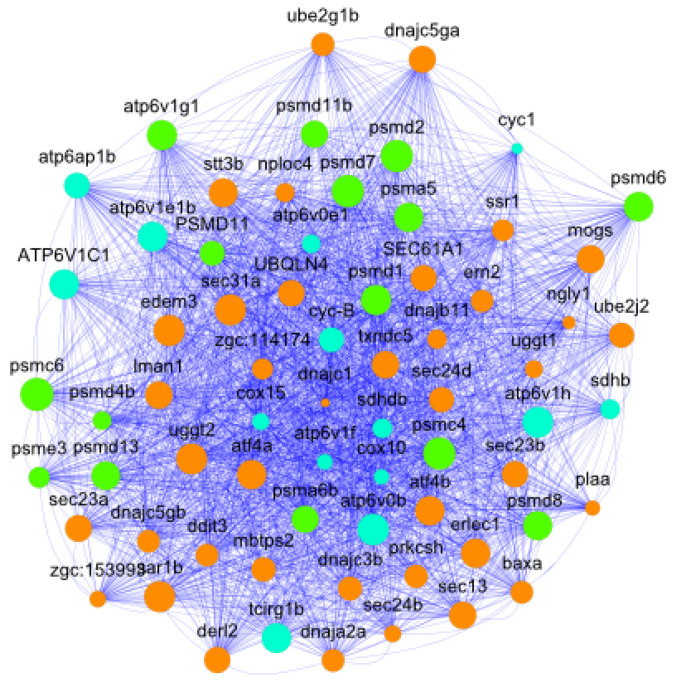
Network visualization of highly connected genes in the MEblue module. Node size is proportional to degree. Orange, green, and cyan nodes represent genes associated with endoplasmic reticulum protein processing, the proteasome, and oxidative phosphorylation, respectively.

The co-expression network of the MEblue module (weight > 0.15) contained 70 genes and was mainly associated with three KEGG pathways: protein processing in the endoplasmic reticulum, the proteasome, and oxidative phosphorylation (Figure 9). Twelve highly connected hub genes were selected for qRT-PCR, including *tcirg1b*, *sec31a*, *sar1b*, and *uggt2* in endoplasmic reticulum protein processing; *psmd1*, *psmd7*, *psmc4*, and *psma5* in the proteasome; and *atp6v0b*, *atp6v1e1b*, *atp6v1g1*, and *ATP6V1C1* in oxidative phosphorylation.

**Figure 10:**
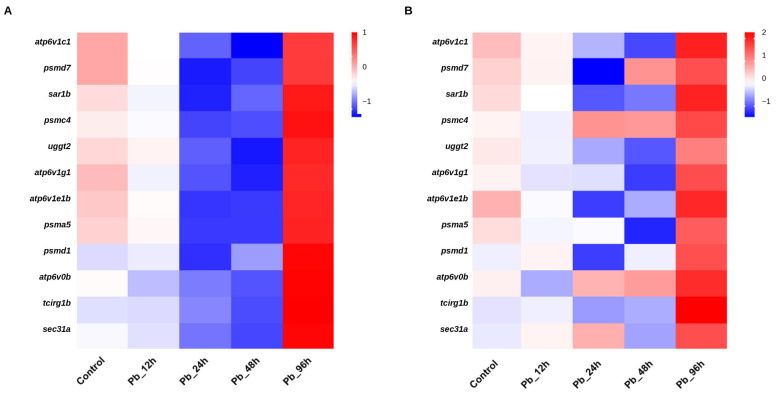
Heatmaps showing expression patterns of the 12 hub genes identified in the MEblue module as measured by RNA-seq (**A**) and qRT-PCR (**B**).

qRT-PCR validation of these 12 hub genes showed high consistency with RNA-Seq results, confirming the reliability of transcriptomic data (Figure 10).

## 4. Discussion

Pb is a persistent environmental contaminant that threatens aquatic organisms by inducing oxidative stress, impairing tissue integrity, and altering transcriptional regulation [4,39,40]. In the present study, juvenile pufferfish exposed to waterborne Pb showed a coordinated hepatic response characterized by Pb accumulation, rapid ROS production, dynamic changes in antioxidant enzymes, progressive lipid peroxidation, and extensive transcriptomic remodeling. These findings support the view that oxidative stress is a central mechanism of Pb-induced hepatotoxicity in fish [10,11,12,13].

Hepatic Pb concentration increased during the exposure period and reached 1.30 μg/g at 96 h, indicating that the liver is an important target organ for Pb retention and detoxification. This observation is consistent with the detoxification role of the fish liver and with previous evidence that metal exposure can lead to hepatic accumulation and tissue injury [14,15,16,17,40]. The absence of a significant difference between the control and 12 h sampling time points may be explained by the relatively short exposure duration and the biological variability among individual fish. The marked increase at 96 h suggests that hepatic retention becomes more pronounced during the late stage of acute exposure.

Pb exposure rapidly induced oxidative stress, as shown by the marked increase in ROS levels in both blood cells and hepatocytes at 12 h. This early ROS burst is consistent with the recognized ability of Pb to promote electron leakage, disrupt antioxidant defense systems, and trigger oxidative damage [4,9,10,11,12,13,41]. The subsequent decline in ROS levels may reflect partial activation of compensatory antioxidant responses, changes in Pb distribution, or adaptive cellular regulation. However, progressive MDA accumulation indicated that lipid peroxidation persisted despite the later decrease in measurable ROS, suggesting that early redox imbalance resulted in sustained membrane oxidative damage [11,12,13,42].

The antioxidant enzyme data further support the occurrence of an acute redox imbalance followed by partial compensation. SOD and CAT activities increased during the early exposure stage, indicating an immediate enzymatic response against superoxide anions and hydrogen peroxide. At 96 h, both enzymes were suppressed, suggesting that prolonged Pb exposure may impair antioxidant capacity or exhaust enzymatic defense. GSH-Px showed a delayed recovery pattern, implying that glutathione-dependent detoxification may contribute to later-stage adaptation. Similar dynamic antioxidant responses have been reported in fish exposed to heavy metals and other waterborne stressors, where initial enzyme activation is followed by inhibition or compensatory recovery depending on exposure intensity and duration [10,11,12,13,43].

Transcriptomic profiling showed that Pb exposure triggered broad and time-dependent reprogramming of hepatic gene expression, with the number of DEGs increasing from 167 at 12 h to 2580 at 96 h. GO and KEGG enrichment results indicated that early responses were related mainly to redox and metabolic regulation, whereas later responses involved lipid metabolism, protein processing, proteasome function, oxidative phosphorylation, and cellular stress pathways. This temporal transition is consistent with studies showing that metal exposure can induce progressive transcriptomic remodeling in fish and other organisms, particularly in pathways related to oxidative stress, metabolism, mitochondrial function, and proteostasis [18,19,20,21,44,45]. Recent RNA-seq studies in zebrafish also demonstrate that acute Pb exposure can alter transcriptional programs associated with development, oxidative stress, cellular structure, and stress adaptation, supporting the relevance of transcriptomic approaches for identifying Pb-responsive pathways [46,47].

STEM analysis provided further temporal resolution and revealed a transition from early stress signaling to later metabolic and proteostasis-related regulation. Enriched pathways included PPAR signaling, steroid biosynthesis, protein processing in the endoplasmic reticulum, the proteasome, cell cycle, apoptosis, and peroxisome pathways. These pathways suggest that Pb exposure not only disrupts antioxidant balance but also affects lipid remodeling, protein quality control, organelle stress, and cell fate regulation. Such pathway-level responses are consistent with recent biomarker-based interpretations of heavy metal toxicity in fish, in which oxidative stress, detoxification, apoptosis, immune regulation, and energy-metabolism disruption are viewed as interconnected molecular outcomes [48].

WGCNA further identified co-expression modules associated with Pb exposure. The application of WGCNA to fish transcriptomic datasets has been increasingly used to identify stress-associated modules and hub genes under environmental or biological challenges [49,50,51,52]. In the present study, the MEblue module was enriched in protein processing in the endoplasmic reticulum, proteasome, and oxidative phosphorylation, highlighting the potential importance of proteostasis and mitochondrial energy metabolism in the hepatic response to Pb exposure. Endoplasmic reticulum stress and proteasomal degradation are closely linked to oxidative damage and the removal of misfolded or damaged proteins, whereas mitochondrial oxidative phosphorylation is directly connected to cellular energy supply and ROS generation [53,54]. Recent summaries of oxidative stress and neurotoxicity biomarkers in fish also support the combined use of ROS, MDA, antioxidant enzymes, and transcriptomic indicators as complementary endpoints for toxicological assessment [55].

The hub genes identified in the MEblue module, including *psmd1*, *psmd7*, *psmc4*, *psma5*, *tcirg1b*, *sec31a*, *sar1b*, *uggt2*, *atp6v0b*, *atp6v1e1b*, *atp6v1g1*, and *atp6v1c1*, were mainly involved in proteasomal degradation, endoplasmic reticulum protein processing, vesicle trafficking, lysosomal acidification, and mitochondrial or vacuolar ATPase-related processes. qRT-PCR validation confirmed expression patterns consistent with RNA-seq results, supporting the reliability of the transcriptomic analysis. These genes may serve as candidate molecular markers for Pb-induced hepatic stress in pufferfish, although functional validation is still required to determine their precise roles in Pb toxicity.

Taken together, the biochemical and transcriptomic results support a time-dependent model of Pb-induced hepatotoxicity. During the early stage, Pb exposure rapidly increases ROS production and activates antioxidant defenses. During prolonged acute exposure, antioxidant capacity becomes insufficient, MDA content accumulates, and transcriptional programs related to metabolism, proteostasis, endoplasmic reticulum stress, proteasome function, and oxidative phosphorylation become increasingly prominent. This integrated response suggests that Pb-induced hepatic toxicity in pufferfish involves both direct oxidative damage and adaptive molecular remodeling.

This study has several limitations. First, although four biological replicates were used at each sampling time point and each replicate was obtained from a different tank, the sample size remains relatively small and may not fully capture the biological variability of pufferfish responses to Pb exposure. Second, this study used a single sublethal Pb concentration and focused on a 96 h acute exposure period; therefore, the results may not fully represent chronic, fluctuating, or environmentally complex Pb exposure scenarios. Third, transcriptomic, STEM, and WGCNA analyses identify candidate pathways and hub genes, but further functional validation is required to confirm their mechanistic roles in Pb-induced hepatotoxicity.

## 5. Conclusions

This study demonstrated that 96 h waterborne Pb exposure induced hepatic Pb accumulation, rapid ROS production, dynamic antioxidant responses, increased MDA content accumulation, and extensive transcriptomic remodeling in juvenile pufferfish. Biochemical assays showed early ROS elevation and antioxidant enzyme activation, followed by late suppression of SOD and CAT activities and persistent lipid peroxidation. RNA-seq, STEM, and WGCNA analyses further revealed time-dependent regulation of metabolic adaptation, protein processing in the endoplasmic reticulum, proteasome function, oxidative phosphorylation, and ATPase-related pathways. qRT-PCR validation supported the reliability of the transcriptomic results. Overall, this study provides an integrated biochemical and transcriptomic framework for understanding Pb-induced hepatic oxidative stress in *T. obscurus* and identifies candidate pathways and hub genes for future mechanistic studies.

## Figures and Tables

**Table 1:** Primers used for qRT-PCR verification of differentially expressed genes.

Gene Name	Forward Primer (5′-3′)	Reverse Primer (5′-3′)
*psmd1*	CTTCCTGTCGCTGGCTTTTAC	CTCCTTCTTCTTGGCTTTGGC
*psmd7*	TCACAACGCATCACAAACCAG	CGTATGAGCGAGGCCAAATAG
*psmc4*	GCTTTCATCCGAGTTGTGGGT	TATAATTGCCGGTGCGTTTTC
*psma5*	TGACAAGATCGGAATATGACAGG	AGAACGACATCGAAAGTGAAAGC
*atp6v0b*	CACCCGCCAAGAAGATACAAAA	CCACAATCGGCAAATAGAGACA
*tcirg1b*	ATGGGCTCCTTGTTCCGCAGTGAGG	CCTCACTGCGGAACAAGGAGCCCAT
*sec31a*	CAATGACACCAGGACCAAAAAC	TGAGAGGAGAGGTAGCAAAACG
*sar1b*	TGTGCCGACTTTACATCCAACC	CCGTACAATCCAAACATTTCCC
*uggt2*	TCTACCAGATCCTCACGCACGAT	GTCCCAGATGCCTTTGCTTTCAC
*atp6v1e1b*	TGGCTTTCAGTTCACACTCTTTT	ACCCCCATTCATCTGTCTATTTC
*atp6v1g1*	GCAACAGCCATCAACACTAAAAAC	AAACCAAGAGAAGAGGGAGAAACA
*atp6v1c1*	TTGCTAACGGAGTTGACCTGGTT	GAGAGGCTTGCTTGGAGATGATT
*gapdh*	GGCCCAATGAAAGGCATTCT	TGGGTGTCGCCGTTGAA
*β-actin*	CATCACCATCGGCAACGAGAGG	CGTCGCACTTCATGATGCTGTTG

Note: Full gene names are as follows: *psmd1*, *proteasome 26S subunit*, *non-ATPase 1*; *psmd7*, *proteasome 26S subunit*, *non-ATPase 7*; *psmc4*, *proteasome 26S subunit*, *ATPase 4*; *psma5*, *proteasome subunit alpha type 5*; *atp6v0b*, *ATPase H+ transporting V0 subunit b*; *tcirg1b*, *T-cell immune regulator 1b*; *sec31a*, *SEC31 homolog A*, *COPII coat complex component*; *sar1b*, *secretion-associated RAS-related GTPase 1B*; *uggt2*, *UDP-glucose glycoprotein glucosyltransferase 2*; *atp6v1e1b*, *ATPase H+ transporting V1 subunit E1b*; *atp6v1g1*, *ATPase H+ transporting V1 subunit G1*; *atp6v1c1*, *ATPase H+ transporting V1 subunit C1*; *gapdh*, *glyceraldehyde-3-phosphate dehydrogenase*; and *β-actin, beta-actin*.

## Data Availability

Raw RNA-seq data have been deposited in the NCBI Sequence Read Archive (SRA) database with the accession numbers PRJNA1284451. The datasets used and analyzed during this current study are available from the corresponding author upon reasonable request.

## Supplementary Materials

The following supporting information can be downloaded at https://www.mdpi.com/article/10.3390/antiox15070827/s1, Figure S1: Functional annotation of all transcripts of pufferfish in six databases: Non-Redundant Protein Sequence Database (NR), Evolutionary Genealogy of Genes: Non-supervised Orthologous Groups (EggNOG), Swiss-Prot Protein Knowledgebase (Swiss-Prot), Protein Families Database (Pfam), Kyoto Encyclopedia of Genes and Genomes (KEGG), Gene Ontology (GO); Table S1: RNA-seq data output and quality-control statistics for each sample. Sample names, raw reads, raw bases, clean reads, clean bases, Q20, Q30, and GC content are shown; Table S2: Functional annotation statistics of transcripts and unigenes in different databases. GO, KEGG, EggNOG, NR, Swiss-Prot, and Pfam annotations are summarized.
