# Supplementary material for: Waterborne Lead Exposure Induces Hepatic Oxidative Stress and Transcriptomic Responses in Pufferfish (Takifugu obscurus)
Source: Antioxidants (Basel). 2026 Jun 30;15(7):827. doi: 10.3390/antiox15070827 (PMC13404945; doi:10.3390/antiox15070827)
Supplement: Supplementary file 1 [file antioxidants-15-00827-s001.zip › antioxidants-4349502-supplementary.pdf]

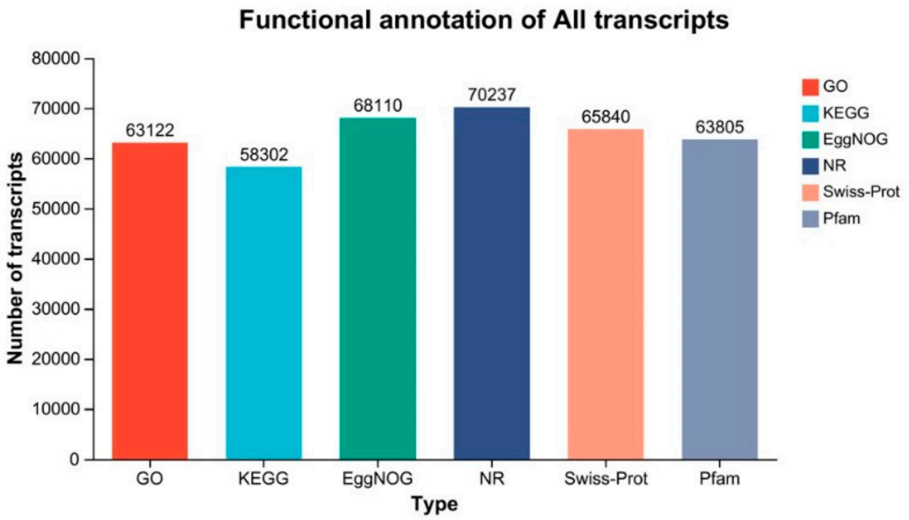


**Figure S1:** Functional annotation of all transcripts of pufferfish in six databases: Non-Redundant Protein Sequence Database (NR), Evolutionary Genealogy of Genes: Non-supervised Orthologous Groups (EggNOG), Swiss-Prot Protein Knowledgebase (Swiss-Prot), Protein Families Database (Pfam), Kyoto Encyclopedia of Genes and Genomes (KEGG), Gene Ontology (GO).

**Table S1:** RNA-seq data output and quality-control statistics for each sample. Sample names, raw reads, raw bases, clean reads, clean bases, Q20, Q30, and GC content are shown.

| Sample | Raw reads | Raw bases | Clean reads | Clean base | Q20 (%) | Q30 (%) |
|---|---|---|---|---|---|---|
| Control_1 | 54 189 972 | 8 182 685 772 | 53 526 528 | 7 869 780 459 | 97.37 | 95.35 |
| Control_2 | 45 531 868 | 6 875 312 068 | 45 025 044 | 6 698 391 381 | 97.34 | 95.21 |
| Control_3 | 54 608 802 | 8 245 929 102 | 54 073 732 | 7 957 944 496 | 97.43 | 95.47 |
| Control_4 | 44 252 568 | 6 682 137 768 | 43 795 236 | 6 499 286 387 | 97.38 | 95.37 |
| Pb_12h_1 | 42 573 306 | 6 428 569 206 | 42 156 956 | 6 258 179 417 | 97.39 | 95.39 |
| Pb_12h_2 | 44 269 690 | 6 684 723 190 | 43 797 190 | 6 480 878 567 | 97.43 | 95.47 |
| Pb_12h_3 | 57 339 038 | 8 658 194 738 | 56 643 126 | 8 338 236 685 | 97.46 | 95.54 |
| Pb_12h_4 | 43 401 154 | 6 553 574 254 | 42 961 794 | 6 377 705 968 | 97.31 | 95.22 |
| Pb_24h_1 | 45 616 322 | 6 888 064 622 | 45 125 830 | 6 710 771 024 | 97.37 | 95.3 |
| Pb_24h_2 | 44 571 164 | 6 730 245 764 | 44 095 796 | 6 549 741 019 | 97.31 | 95.24 |
| Pb_24h_3 | 41 329 688 | 6 240 782 888 | 40 918 164 | 6 090 299 404 | 97.34 | 95.28 |
| Pb_24h_4 | 49 690 932 | 7 503 330 732 | 49 152 516 | 7 302 331 608 | 97.36 | 95.33 |
| Pb_48h_1 | 49 788 068 | 7517998 268 | 49 275 128 | 7 229 663 190 | 97.46 | 95.53 |
| Pb_48h_2 | 50 223 936 | 7583814336 | 49 719 408 | 7 311 718 783 | 97.43 | 95.48 |
| Pb_48h_3 | 53 293 870 | 8047374370 | 52 723 810 | 7 727 208 308 | 97.44 | 95.52 |
| Pb_48h_4 | 51 227 286 | 7735320186 | 50 721 068 | 7 487 370 882 | 97.48 | 95.57 |
| Pb_96h_1 | 53 483 946 | 8076075846 | 53 011 968 | 7 850 172 129 | 97.57 | 95.73 |
| Pb_96h_2 | 45 423 766 | 6858988666 | 44 970 430 | 6 668 842 855 | 97.38 | 95.36 |
| Pb_96h_3 | 45 811 660 | 6917560660 | 45 380 978 | 6 724 205 387 | 97.48 | 95.56 |
| Pb_96h_4 | 45 019 028 | 6797873228 | 44 540 782 | 6 601 494 211 | 97.32 | 95.26 |

**Table S2:** Functional annotation statistics of transcripts and unigenes in different databases. GO, KEGG, EggNOG, NR, Swiss-Prot, and Pfam annotations are summarized.

| Database | Number of Transcripts | Percentage (%) | Number of Unigenes | Percentage (%) |
|---|---|---|---|---|
| GO | 63 122 | 86.90 | 21 720 | 84.75 |
| KEGG | 58 302 | 80.27 | 18 610 | 72.62 |
| EggNOG | 68 110 | 93.77 | 22 762 | 88.82 |
| NR | 70 237 | 96.70 | 24 103 | 94.05 |
| Swiss-Prot | 65 840 | 90.65 | 21 607 | 84.31 |
| Pfam | 63 805 | 87.84 | 20 291 | 79.18 |
| Total_anno | 70 645 | 97.26 | 24 463 | 95.46 |
| Total | 72 634 | 100 | 25 627 | 100 |
